# Multilayer Lateral Heterostructures of Van Der Waals Crystals with Sharp, Carrier–Transparent Interfaces

**DOI:** 10.1002/advs.202103830

**Published:** 2021-11-23

**Authors:** Eli Sutter, Raymond R. Unocic, Juan‐Carlos Idrobo, Peter Sutter

**Affiliations:** ^1^ Department of Mechanical & Materials Engineering and Nebraska Center for Materials and Nanoscience University of Nebraska‐Lincoln Lincoln NE 68588 USA; ^2^ Center for Nanophase Materials Sciences Oak Ridge National Laboratory Oak Ridge TN 37831 USA; ^3^ Department of Electrical & Computer Engineering University of Nebraska‐Lincoln Lincoln NE 68588 USA

**Keywords:** germanium sulfide, interfaces, lateral heterostructures, layered semiconductors, optoelectronics, tin sulfide, van der Waals crystals

## Abstract

Research on engineered materials that integrate different 2D crystals has largely focused on two prototypical heterostructures: Vertical van der Waals stacks and lateral heterostructures of covalently stitched monolayers. Extending lateral integration to few layer or even multilayer van der Waals crystals could enable architectures that combine the superior light absorption and photonic properties of thicker crystals with close proximity to interfaces and efficient carrier separation within the layers, potentially benefiting applications such as photovoltaics. Here, the realization of multilayer heterstructures of the van der Waals semiconductors SnS and GeS with lateral interfaces spanning up to several hundred individual layers is demonstrated. Structural and chemical imaging identifies {110} interfaces that are perpendicular to the (001) layer plane and are laterally localized and sharp on a 10 nm scale across the entire thickness. Cathodoluminescence spectroscopy provides evidence for a facile transfer of electron‐hole pairs across the lateral interfaces, indicating covalent stitching with high electronic quality and a low density of recombination centers.

## Graphical abstract

Multilayer heterostructures with localized and sharp lateral interfaces across hundreds of individual van der Waals layers are realized from the anisotropic layered semiconductors SnS and GeS. Cathodoluminescence spectroscopy shows the transfer of electron–hole pairs across the lateral interfaces, demonstrating excellent connectivity without interfacial recombination. The results are promising for applications benefiting from thick layered crystals combined with in‐plane carrier manipulation.

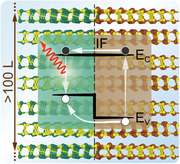

## 1 Introduction

The advent of 2D crystals has opened up exceptional opportunities for materials integration.^[^ ^1^ ^]^ Vertical van der Waals stacks, providing electronic hybridization,^[^ ^2^ ^]^ interlayer excitons,^[^ ^3^ ^]^ as well as twist effects,^[^ ^4^ ^]^ are usually assembled by micromechanical stacking of exfoliated sheets. Lateral heterostructures, realizing carrier manipulation at covalent line interfaces, are only accessible via bottom‐up synthesis. Growth processes for lateral integration have been developed for different 2D crystals, including graphene‐hBN^[^ ^5^ ^]^ and several transition metal dichalcogenides.^[^ ^6^, ^7^ ^]^ Limited so far to monolayers, the concept of lateral heterostructures could be extended to few‐layer^[^ ^8^ ^]^ and even multilayer van der Waals crystals,^[^ ^9^ ^]^ e.g., to combine intralayer carrier manipulation with an enhanced optical thickness and emerging photonic properties.^[^ ^10^ ^]^ Here, we discuss multilayer lateral heterostructures integrating SnS and GeS, two anisotropic van der Waals semiconductors. In a two‐step process, thick SnS seed crystals are synthesized by vapor transport, exposed to GeS and thereby converted into heterostructures with abrupt lateral interfaces across hundreds of layers. Nanoscale spectroscopy shows efficient transfer of electron‐hole pairs across these interfaces. The findings provide a fresh perspective on materials integration beyond the archetypal monolayer heterostructures of 2D crystals.

## 2 Results and Discussion

**Figure 1:**
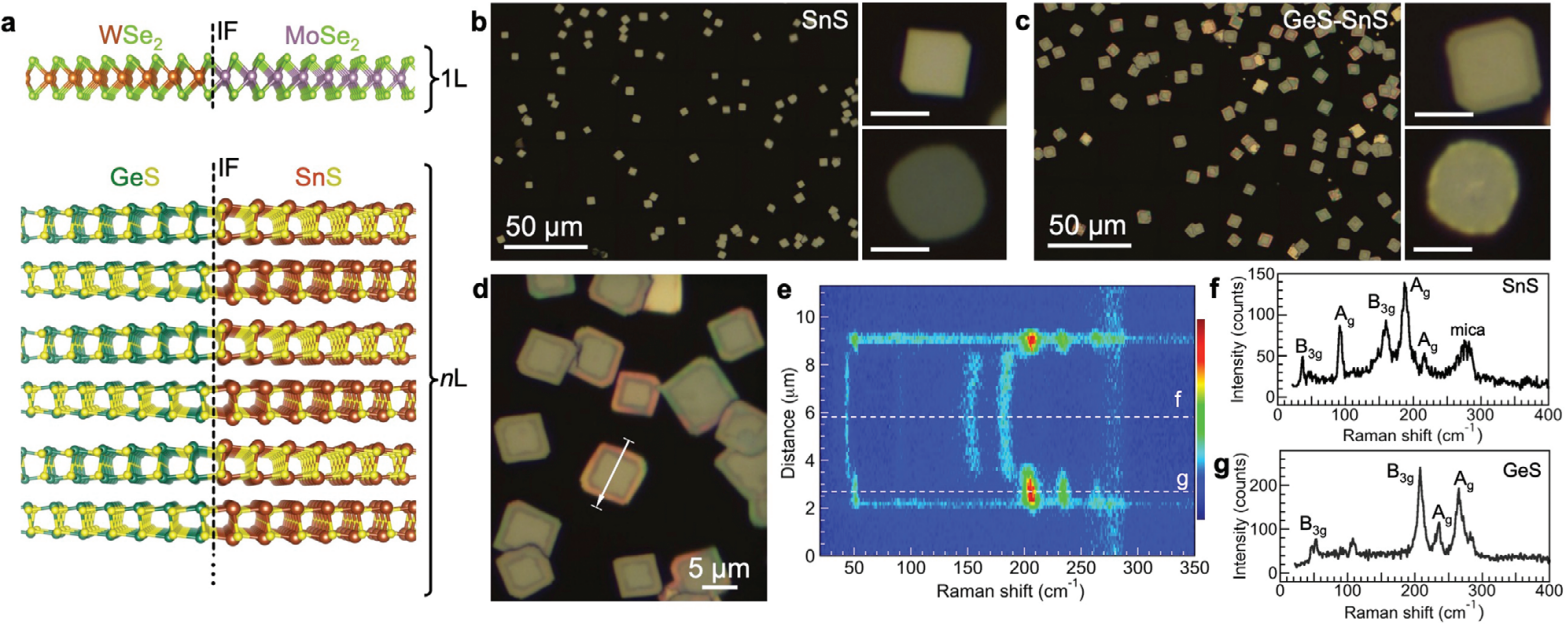
Bottom‐up synthesis of multilayer lateral heterostructures of van der Waals crystals. a) Conventional lateral heterostructure between monolayer MoSe_2_ and WSe_2_ (top) and multilayer lateral heterostructure (bottom) in which each of many van der Waals layers contains an interface, here between orthorhombic GeS and SnS. b) Optical microscopy of typical SnS seed flakes grown on mica substrates. Scale bars in zoomed‐in images: 5 µm. c) Optical images of the sample shown in b. following GeS growth at GeS precursor temperature of 420 °C, which results in the formation of GeS–SnS heterostructures. Zoomed‐in images show characteristic optical contrast between the SnS core and GeS edge rim in thick {110} faceted heterostructures (scale bars: 5 µm). d) Typical ensemble of multilayer GeS–SnS heterostructures. e) Raman linescan across the GeS–SnS heterostructure shown in d. f) Raman spectrum obtained in the center of the heterostructure (dashed line "f" in e.), showing SnS vibrational modes. g) Raman spectrum from the periphery (dashed line "g" in e.), showing GeS vibrational modes.

Multilayer GeS–SnS heterostructures (**Figure** **1**a) were prepared via a two‐step growth process (see Experimental Section).^[^ ^9^ ^]^ Large SnS seeds were grown on mica under conditions favoring thick (>25 nm) flakes bounded by long straight {110} side facets (Figure 1b).^[^ ^11^, ^12^ ^]^ Occasional thinner flakes show rounded shapes, consistent with previous results.^[^ ^11^ ^]^ Subsequent GeS growth leads to a significant increase in the lateral flake size while maintaining faceted shapes (Figure 1c; Figure S1, Supporting Information), consistent with GeS attachment to the lateral edges of the SnS seeds. Optical images confirm this scenario, showing contrast between the centers and the edge rim (Figure 1d) while Raman line scans (Figure 1e) detect SnS^[^ ^13^ ^]^ and GeS^[^ ^14^, ^15^, ^16^ ^]^ vibrational modes in the center and edge regions, respectively (Figure 1f,g).

**Figure 2:**
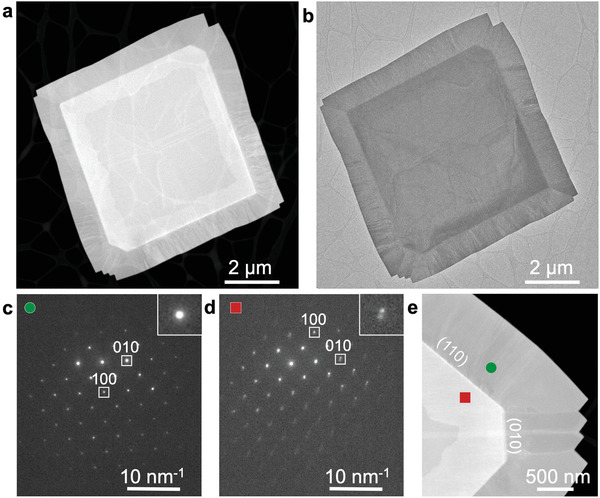
Structure and morphology of multilayer GeS–SnS heterostructures. a) HAADF‐STEM, b) TEM image of a characteristic GeS–SnS heterostructure (GeS precursor temperature: 420 °C). c) Nanobeam electron diffraction pattern obtained on the GeS side of the multilayer lateral interface (green dot in e.). d) Nanobeam electron diffraction pattern from the central region of the heterostructure (red square in e). The diffraction patterns from the periphery demonstrate single spots consistent with GeS, while the central region shows double (010) spots due to the ***a***‐axis mismatch between GeS and SnS. The (100) reflections coincide, consistent with the small ***b***‐axis mismatch. Zone axis (ZA): [001]. e) Higher magnification STEM image of the interface, showing GeS grains and vertical grain boundaries with faceted grooving along the short (010) facet segment due to the large ***a***‐axis mismatch (see also Figures S2 and S3, Supporting Information).

Structure and morphology of the heterostructures were investigated by (scanning) transmission electron microscopy ((S)TEM) and electron diffraction. Following the two‐step growth, typical flakes (**Figure** **2**a,b) have faceted edges as observed in optical images with the central SnS region showing a kinetic growth shape with large straight {110} facets, minor {010} facets, and sharp corners along 〈100〉 directions.^[^ ^17^ ^]^ Nanobeam electron diffraction (Figure 2c,d) identifies the peripheral band as single‐crystalline GeS^[^ ^18^ ^]^ imaged along the [001] zone axis, while the center shows a superposition of single‐crystal diffraction patterns of vertically stacked SnS^[^ ^19^ ^]^ and GeS with aligned lattices.^[^ ^9^ ^]^ GeS and SnS have the same orthorhombic structure. In the plane, the lattice mismatch is negligible (≈0.3%) along [010] (***b***), with ***b***_SnS_ = 0.4443 nm and ***b***_GeS_ = 0.4455 nm, but is large (≈8.9%) along [100] (***a***), with ***a***_SnS_ *=* 0.4024 nm and ***a***_GeS_ *=* 0.3666 nm (Figure S2, Supporting Information). Along the {110} side facets, the mismatch (averaging ≈3.9%) is relaxed by misfit dislocations^[^ ^9^ ^]^ but the GeS band connects seamlessly with the SnS edge and is single crystalline. In the cut‐off corner regions with extended {010} facets, the large ***a***‐axis mismatch causes more severe effects (Figure 2e, Figures S2 and S3, Supporting Information) that range from polycrystalline GeS growth with clearly visible vertical grain boundaries and edge grooving to entirely suppressed local GeS growth.

The second growth step results in GeS attachment to the edges of the SnS seeds and formation of a multilayer lateral heterostructure. In addition, diffraction (Figure 2d) shows a thin GeS capping layer across the central SnS region. GeS covers the entire top surface, albeit with varying thickness. Atomic force microscopy (Figure S4, Supporting Information) confirms a thicker GeS cap near the lateral interface, consistent with a band of brighter contrast observed in HAADF‐STEM (Figure 2a,e). Sequential growth thus produces heterostructures that host covalent multilayer lateral interfaces between SnS and GeS along with a vertical van der Waals interface between the SnS seed and a few‐layer GeS cap.

**Figure 3:**
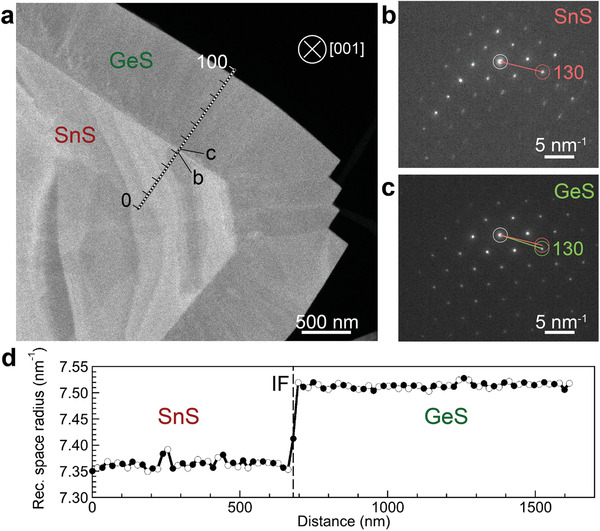
Nanobeam diffraction analysis across the lateral interface in multilayer GeS–SnS heterostructures. a) Plan‐view HAADF‐STEM image of a representative multilayer lateral GeS–SnS heterostructure with small thickness of the GeS cap layer across the SnS center. Along the marked line, 100 nanobeam electron diffraction patterns were obtained across the lateral interface. b) Nanobeam diffraction pattern obtained on the SnS side of the lateral interface (position "b" in panel a). c) Nanobeam diffraction pattern obtained on the GeS side of the lateral interface (position "c" in panel a). d) Line profile of the reciprocal spacing between the zone center (white circles in b,c) and the (130) diffraction spot. Note the abrupt transition between SnS and GeS reciprocal vectors across the lateral interface (IF).

**Figure** **3** shows a nanobeam electron diffraction analysis of the local lattice parameters across the lateral interface, which provides a crystallographic measure of the sharpness of the interface. A series of nanobeam diffraction patterns was obtained in plan view along a line crossing the lateral interface (Figure 3a), and the transition between SnS and GeS was tracked by measuring the reciprocal space distance of the (130) diffraction spot from the zone center. Individual nanobeam diffraction patterns obtained in close proximity on either side of the interface show pure SnS (Figure 3b) and GeS (Figure 3c) structure, respectively. An analysis of the 130 spot position in all diffraction patterns, shown in Figure 3d, illustrates the sharp transition between SnS and GeS at the lateral multilayer interface over a distance of ≈15 nm (80:20 criterion applied to Figure 3d). In contrast to monolayer lateral heterostructures, where the sharpness of a straight line interface is determined purely by alloying of the joined components, a multilayer heterostructure has two possible contributions that can lead to a broadening of the interface: Alloying and delocalization, i.e., misalignment between the line interfaces in the different van der Waals layers (Figure S5, Supporting Information). The crystallographic analysis of Figure 3 shows that the combined effect of these two contributions causes very limited lateral broadening of the interface on a ≈10 nm scale.

**Figure 4:**
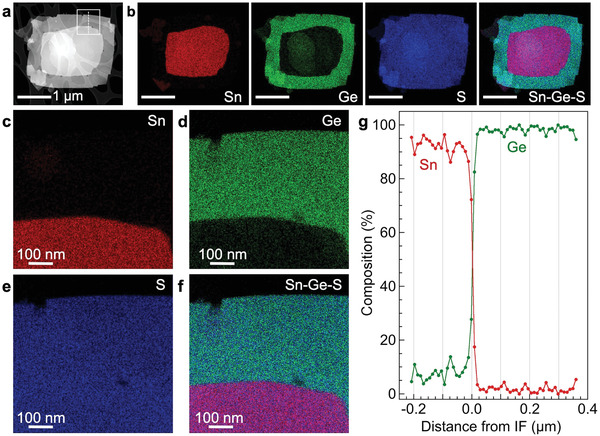
Chemical imaging of multilayer GeS–SnS heterostructures. a) HAADF‐STEM image of a representative multilayer lateral GeS–SnS heterostructure. b) EDS maps showing the distribution of Sn (red), Ge (green), S (blue), along with an overlay map of the heterostructure. c–f) Higher magnification EDS chemical maps of the interfacial region (square in a). **g)** EDS line profile across the lateral interface (IF, dashed line in a), showing the cation (Sn (red); Ge (green)) distribution in the interfacial region. The measured interface width is ≈11 nm (80:20 criterion). The GeS cap layer across the SnS flake (left) amounts to ≈6% of the total thickness.

Chemical analysis by energy‐dispersive X‐ray spectroscopy (EDS) elemental maps and line scans (**Figure** **4**) in plan‐view geometry provides a complementary characterization of the interface by identifying the distribution of Ge, Sn, and S in the GeS–SnS heterostructures. Ge is detected in the edge region as well as in smaller amounts in the core due to the thin GeS cap layer across the SnS seed. Sn is limited to the footprint of the SnS seed flake. The S signal is quite uniform over the entire heterostructure, consistent with a constant 1:1 ratio of S to the Ge/Sn cations. In high‐magnification elemental maps (Figure 4c–f) the multilayer lateral interface between GeS and SnS appears very sharp. The interface width has been quantified in EDS line scans (Figure 4g), which show typical transitions (e.g., from 80% to 20% Ge content) over ≈11 nm. This finding has two important implications. Firstly, it indicates negligible alloying between SnS and GeS near the lateral interfaces. This is perhaps not surprising since GeS is grown at temperatures where SnS is thermally stable,^[^ ^17^ ^]^ but our prior work did show significant interfacial alloying, especially for thinner heterostructures.^[^ ^9^ ^]^ Second, the sharp transition in composition demonstrates the near‐perfect alignment of the lateral interfaces across hundreds of individual van der Waals layers (see Figure S5 in the Supporting Information). This vertical alignment of the individual line interfaces is a consequence of the pronounced {110} edge faceting of the SnS seeds, which is preserved due to the absence of intermixing during the second growth step. Comparison of EDS spectra obtained within the central SnS region, the GeS edge band, as well as in the area of the lateral interface (Figure S6, Supporting Information) shows the absence of contaminants (notably oxygen) at the interface, indicating seamless covalent stitching. Further evidence for seamless connectivity at the lateral interfaces is provided by cathodoluminescence spectroscopy (see Figure 6). The absence of oxidation at the edges of the first‐grown SnS seeds despite transfer through air is likely due to the formation of a thin S‐rich protective shell surrounding the SnS flakes,^[^ ^13^ ^]^ which is reduced during the subsequent GeS growth step at elevated temperature.

**Figure 5:**
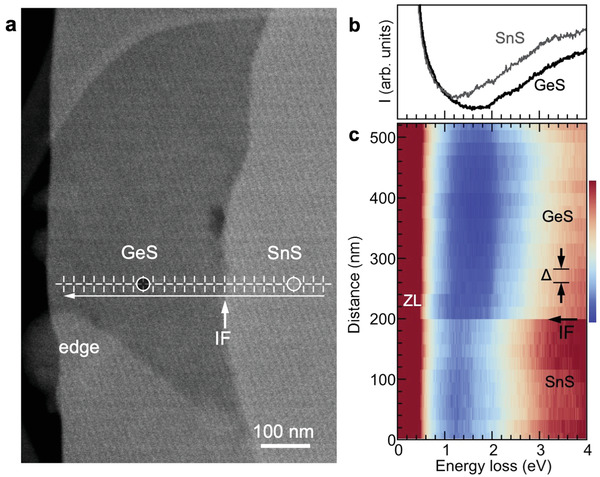
Nanometer‐scale absorption measurements by valence EELS. a) HAADF‐STEM image of a multilayer heterostructure close to the interface between the GeS periphery and the SnS core. IF: Lateral interface between SnS and GeS. b) Monochromated STEM‐EELS spectra obtained at points on the SnS and GeS side of the lateral interface (circles in a). c) Monochromated STEM‐EELS spectrum line scan (spatial step Δ = 20 nm) comprising full EEL spectra measured across the lateral interface between multilayer SnS and GeS (at crosshair points shown in a). IF: Position of the interface. ZL: Zero‐loss peak.

The optoelectronic properties of GeS–SnS multilayer lateral heterostructures were probed with nanometer resolution by techniques employing focused electron beam excitation in STEM. Local absorption measurements used high‐resolution electron energy‐loss spectroscopy (EELS) in monochromated, aberration‐corrected STEM (Figure S7, Supporting Information). Luminescence was probed by cathodoluminescence spectroscopy in STEM (STEM‐CL). Absorption measurements (**Figure** **5**) show clear differences in the energy‐loss onset associated with interband transitions in the GeS and SnS parts of the heterostructure, consistent with the larger fundamental gap in GeS (*E*_g_ = 1.65 eV) compared to SnS (***a***‐ and ***b***‐valley bandgaps in bulk SnS: 1.35 eV and 1.55 eV, respectively).^[^ ^20^ ^]^ The EELS measurements are local, i.e., the losses represent electronic transitions at the position of the focused electron beam. The EELS line scan demonstrates a sharp transition between GeS and SnS across the multilayer interface (Figure 5a), consistent with the abrupt changes in structure and composition detected by nanobeam diffraction (Figure 3) and EDS (Figure 4), respectively.

**Figure 6:**
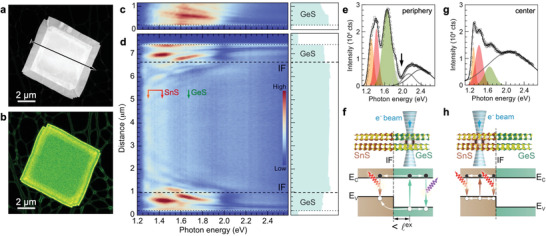
Characterization of charge transfer across the lateral interface by nanoscale STEM‐CL luminescence spectroscopy. a) HAADF‐STEM image of a GeS–SnS multilayer heterostructure. b) Corresponding panchromatic STEM‐CL map (wavelength range: 400 nm ≤ *λ* ≤ 1000 nm). c) Hyperspectral STEM‐CL line scan of a homogeneous multilayer GeS reference flake. d) Hyperspectral STEM‐CL linescan of a multilayer SnS–GeS heterostructure (measured along arrow in a), with axis scaling identical to that in c. Dotted and dashed horizontal lines mark the flake edges and lateral interfaces (IF), respectively. Dispersive emission features in the GeS bands are due to interference of photonic waveguide modes.^[^ ^21^ ^]^ Arrows mark the energy of SnS and GeS band‐edge emissions. e) CL spectrum obtained in the peripheral GeS region of the heterostructure. Note the apparent SnS luminescence character for excitation in the GeS region of the heterostructure, indicative of electron–hole pair transfer from GeS to SnS across the multilayer lateral interface. Arrow: Quenching of the higher‐energy (≈1.9 eV) luminescence. f) Schematic of the local electron‐beam excitation in the peripheral GeS region of the heterostructures: Emitted light originates from radiative recombination in GeS as well as recombination of electron–hole pairs transmitted across the interface into SnS. g) CL spectrum obtained in the central SnS region of the heterostructure. Shaded peaks in e,f represent a Gaussian lineshape analysis showing two SnS emissions at low and one GeS emission at higher photon energy. h) Electron‐beam excitation within the SnS part of the heterostructures: The step in the valence band precludes hole transfer from SnS to GeS, i.e., the emitted light originates only from radiative recombination in SnS (see also Figure S10, Supporting Information).

A complementary nonlocal picture of optoelectronic excitations is provided by STEM‐CL, using a focused (≈1–2 nm) electron beam as an excitation source while detecting emitted light in the far field.^[^ ^14^ ^]^ Since excited states such as electron–hole pairs (or excitons) can travel some distance from the point of excitation before recombining, STEM‐CL lends itself uniquely to probing excitation transfer across the multilayer lateral interfaces in our heterostructures (**Figure** **6**). Panchromatic CL mapping shows bright light emission overall (Figure 6a,b). Full CL spectra were obtained in line scans across individual multilayer GeS–SnS heterostructures, as well as reference samples consisting of homogeneous multilayer GeS flakes of similar thickness (without an interface). Pure GeS flakes (Figure 6c) show characteristic dispersive fringes due to interference of edge‐reflected waveguide modes,^[^ ^21^ ^]^ which rapidly lose intensity below 1.6 eV photon energy. Similar fringes are seen in the GeS band near the edge of the heterostructure, as well as in the SnS center where they transition into two distinct band‐edge luminescence peaks at 1.3 eV and 1.45 eV, respectively (Figure 6d, see also Figures S8 and S9, Supporting Information). The center region also shows weak emission at higher energy originating from the thin GeS cap layer.

Clear signatures of carrier transfer across the multilayer lateral interface are seen in the CL excited in the peripheral GeS band of the heterostructures (Figure 6d–f). Aside from the characteristic GeS band‐edge luminescence at ≈1.65 eV photon energy,^[^ ^21^ ^]^ electron beam excitation in the GeS region produces additional low‐energy emission that is not seen in the homogeneous GeS reference sample (Figure 6c), notably in the form of two intense peaks at photon energies between 1.5 and 1.3 eV (i.e., coinciding with SnS interband transitions). The emergence of these low‐energy emission peaks is accompanied by a pronounced quenching of the luminescence at ≈1.9 eV, which is also absent in spectra of the homogeneous GeS reference sample (Figure 6c–e). On the other hand, positioning the exciting electron beam on the SnS side of the lateral interface gives rise to two predominant peaks originating from the ***a***‐ and ***b***‐valleys in SnS,^[^ ^20^ ^]^ with only a small higher‐energy peak originating from the thin GeS cap layer (Figure 6g‐h).

The observation of characteristic SnS luminescence resulting from local electron beam excitation on the GeS side of the lateral interface suggests that a significant fraction of electron‐hole pairs excited by the electron beam in GeS are transferred across the interface into SnS before recombining radiatively. This scenario is consistent with calculated band offsets between unstrained GeS and SnS^[^ ^22^ ^]^ (Figure 6f,h, Figure S10, Supporting Information), where a step in the valence band and aligned conduction band edges enables the transfer of intact electron–hole pairs across the lateral interface from GeS to SnS, in contrast to the frequently observed carrier separation at type II heterointerfaces.^[^ ^6^, ^13^ ^]^ The observation of this electron–hole pair transfer indicates high‐quality interfaces that are essentially free of deep‐level recombination centers and therefore are highly transparent to carrier flow within the individual GeS–SnS heterolayers.

## 3 Conclusions

In conclusion, we have demonstrated multilayer lateral heterostructures of van der Waals crystals with interfaces that are abrupt and spatially coordinated over many individual layers, synthesized by vapor transport growth of SnS seeds followed by edge attachment of GeS. In addition, the high surface reactivity of the monochalcogenides^[^ ^23^ ^]^ promotes the growth of a thin GeS capping layer that is van der Waals stacked over the SnS seed. While an unusual band alignment precludes charge separation at the SnS–GeS interface, the covalently stitched multilayer lateral interfaces are highly transparent to the transfer of intact electron–hole pairs (excitons) from GeS into SnS within the individual layers, i.e., without crossing any van der Waals gaps. Demonstrated here for a particular materials system, the concept of lateral heterostructures of multilayer van der Waals crystals could be extended to other layered materials, including a broad range of transition metal dichalcogenides, black phosphorus, etc., provided that the challenge of obtaining vertically faceted, planar multilayer seed crystals can be overcome^[^ ^24^ ^]^ and subject to structural and lattice‐matching compatibility requirements for the components.^[^ ^6^, ^7^ ^]^ Possible future directions in harnessing the functionality provided by multilayer lateral interfaces include the tuning of band offsets to achieve efficient charge separation for photovoltaics,^[^ ^13^, ^25^ ^]^ the realization of lateral interfacial excitons,^[^ ^26^ ^]^ and the study of polariton transport^[^ ^10^ ^]^ across and along interfaces, among others.

## 4 Experimental Section

### Growth Step I—SnS Seed Flakes on Mica

Large multilayer SnS seed crystals were synthesized in a quartz tube reactor with a single temperature‐controlled zone. SnS source powder (99.99%, Sigma Aldrich) in a quartz boat was placed in the center of the heated zone. Freshly cleaved mica substrates (MTI Crystal) supported on Si were placed 10–12 cm from the SnS source. Following pump‐down to <10^−3^ Torr, an Ar/H_2_ (ratio 98:2) carrier gas was introduced at 60 standard cubic centimeters per minute (sccm) flow rate and a pressure of 76 Torr. The use of an evacuated reactor and inert carrier gas ensures low concentrations of oxygen and other contaminants during the growth process. The temperature of the heated zone was increased to 650 °C over 30 min and maintained at this temperature for 5 min. The reactor was then evacuated to <10^−3^ Torr and naturally cooled to room temperature.

### Growth Step II—GeS–SnS Heterostructure Formation

GeS was deposited on the SnS seeds via vapor transport from GeS powder (99.99%, Sigma Aldrich) in a reactor with two independently controlled temperature zones. The evaporation zone containing a quartz boat with GeS powder (≈25 mg) was heated to temperatures between 400 and 420 °C, while the zone containing the SnS seeds on mica was heated to 300–320 °C. During growth an Ar/H_2_ (ratio 98:2) carrier gas flow was maintained at 60 sccm and 76 Torr pressure. GeS growth was performed for 5–8 min, after which the reactor was cooled down naturally.

### Optical Microscopy, Raman Spectroscopy and Mapping

Optical microscopy and micro‐Raman spectroscopy/mapping were performed in air in an optical/Raman microscope (Horiba Xplora plus). Optical imaging employed a 100 × objective and image stitching to cover large sample areas. Raman spectroscopy was performed with a 100 × objective at 532 nm excitation wavelength and 16.8 µW laser power. Raman spectra and line scans were measured using a 300 µm pinhole at ≈0.5 µm spatial resolution.

### Electron Microscopy and Nanobeam Diffraction

Structure and morphology of the heterostructures were investigated by (scanning) transmission electron microscopy ((S)TEM) and nanobeam electron diffraction in an FEI Talos F200X field emission microscope. GeS–SnS heterostructures were transferred from mica substrates to TEM grids using stabilization by spin‐coated (3000 rpm, 60 s) poly(methyl methacrylate) (PMMA) films, baked at 70 °C for 5 min, followed by release of the heterostructure/PMMA sandwich by de‐ionized water penetration.^[^ ^16^ ^]^ After pickup by a TEM grid the PMMA film was dissolved by immersion in acetone, leaving GeS–SnS heterostructures on the grid.

### EDS in STEM

STEM‐EDS maps (1024 × 1024 pixels) and EDS line scans (13 nm step size) were collected using a JEOL NeoARM S/TEM operating at 80 kV.

### Electron Energy Loss Spectroscopy (EELS)

Absorption was measured by valence EELS in a monochromated aberration‐corrected STEM (Nion Hermes at Oak Ridge National Laboratory) operated at 60 kV. Spectra were acquired with an energy resolution of about 60 meV, as measured by the full‐width half‐maximum (FWHM) of the zero‐loss peak (see Figure S7 in the Supporting Information).

### Cathodoluminescence Spectroscopy

Cathodoluminescence spectroscopy was performed in STEM (STEM‐CL) using a Gatan Vulcan CL holder at room temperature, 200 keV electron energy, and incident beam currents of 300–600 pA. Panchromatic CL maps (512 × 512 pixels, 1.28 ms per pixel) were acquired by scanning the exciting electron beam and recording the emitted light intensity over a broad wavelength range (400–1000 nm). Hyperspectral linescans were acquired by displacing the electron beam in equal steps across individual heterostructures and acquiring full CL spectra (integration time: 10 s per spectrum) at each beam position.

## Conflict of Interest

The authors declare no conflict of interest.

## Author Contributions

P.S. and E.S. devised the study, performed electron microscopy, nanobeam diffraction, and cathodoluminescence spectroscopy, jointly analyzed the data and wrote the paper. R.R.U. carried out the STEM‐EDS composition analysis. J.‐C.I. performed valence‐EELS measurements. All authors read and commented on the manuscript.

## Supporting information

Supporting Information

## Data Availability

Research data are not shared.

## References

[1] A. K. Geim , I. V. Grigorieva , Nature 2013, 499, 419. 23887427 10.1038/nature12385

[2] H. Fang , C. Battaglia , C. Carraro , S. Nemsak , B. Ozdol , J. S. Kang , H. A. Bechtel , S. B. Desai , F. Kronast , A. A. Unal , G. Conti , C. Conlon , G. K. Palsson , M. C. Martin , A. M. Minor , C. S. Fadley , E. Yablonovitch , R. Maboudian , A. Javey , Proc. Natl. Acad. Sci. USA 2014, 111, 6198. 24733906 10.1073/pnas.1405435111 PMC4035947

[3] a) E. V. Calman , M. M. Fogler , L. V. Butov , S. Hu , A. Mishchenko , A. K. Geim , Nat. Commun. 2018, 9, 1895; 29760404 10.1038/s41467-018-04293-7 PMC5951911

[4] a) Y. Cao , V. Fatemi , S. Fang , K. Watanabe , T. Taniguchi , E. Kaxiras , P. Jarillo‐Herrero , Nature 2018, 556, 43; 29512651 10.1038/nature26160

[5] a) M. P. Levendorf , C.‐J. Kim , L. Brown , P. Y. Huang , R. W. Havener , D. A. Muller , J. Park , Nature 2012, 488, 627; 22932386 10.1038/nature11408

[6] a) X. Duan , C. Wang , J. C. Shaw , R. Cheng , Y. Chen , H. Li , X. Wu , Y. Tang , Q. Zhang , A. Pan , J. Jiang , R. Yu , Y. Huang , X. Duan , Nat. Nanotechnol. 2014, 9, 1024; 25262331 10.1038/nnano.2014.222 PMC12049235

[7] a) C. Huang , S. Wu , A. M. Sanchez , J. J. P. Peters , R. Beanland , J. S. Ross , P. Rivera , W. Yao , D. H. Cobden , X. Xu , Nat. Mater. 2014, 13, 1096; 25150560 10.1038/nmat4064

[8] P. K. Sahoo , S. Memaran , F. A. Nugera , Y. Xin , T. Díaz Márquez , Z. Lu , W. Zheng , N. D. Zhigadlo , D. Smirnov , L. Balicas , H. R. Gutiérrez , ACS Nano 2019, 13, 12372. 31532628 10.1021/acsnano.9b04957

[9] E. Sutter , J. Wang , P. Sutter , ACS Nano 2020, 14, 12248. 32886477 10.1021/acsnano.0c05978

[10] F. Hu , Y. Luan , M. E. Scott , J. Yan , D. G. Mandrus , X. Xu , Z. Fei , Nat. Photonics 2017, 11, 356.

[11] E. Sutter , J. Wang , P. Sutter , Chem. Mater. 2020, 32, 8034.

[12] P. Sutter , H. P. Komsa , H. Lu , A. Gruverman , E. Sutter , Nano Today 2021, 37, 101082.

[13] P. Sutter , J. Wang , E. Sutter , Adv. Mater. 2019, 31, 1902166. 10.1002/adma.201902166 31157467

[14] P. Sutter , S. Wimer , E. Sutter , Nature 2019, 570, 354. 31011183 10.1038/s41586-019-1147-x

[15] a) H. R. Chandrasekhar , R. G. Humphreys , M. Cardona , Phys. Rev. B 1977, 16, 2981;

[16] E. Sutter , B. Zhang , M. Sun , P. Sutter , ACS Nano 2019, 13, 9352. 31305983 10.1021/acsnano.9b03986

[17] E. Sutter , J. Wang , P. Sutter , Chem. Mater. 2019, 31, 2563.

[18] H. Wiedemeier , H. G. V. Schnering , Z. Kristallogr. 1978, 148, 295.

[19] A. Jain , S. P. Ong , G. Hautier , W. Chen , W. D. Richards , S. Dacek , S. Cholia , D. Gunter , D. Skinner , G. Ceder , K. Persson , APL Mater. 2013, 1, 011002.

[20] S. Lin , A. Carvalho , S. Yan , R. Li , S. Kim , A. Rodin , L. Carvalho , E. M. Chan , X. Wang , A. H. Castro Neto , J. Yao , Nat. Commun. 2018, 9, 1455. 29654301 10.1038/s41467-018-03897-3 PMC5899090

[21] P. Sutter , C. Argyropoulos , E. Sutter , Nano Lett. 2018, 18, 4576. 29883126 10.1021/acs.nanolett.8b01840

[22] B. D. Malone , E. Kaxiras , Phys. Rev. B 2013, 87, 245312.

[23] P. Sutter , E. Sutter , ACS Appl. Nano Mater. 2018, 1, 3026.

[24] J. Zheng , X. Yan , Z. Lu , H. Qiu , G. Xu , X. Zhou , P. Wang , X. Pan , K. Liu , L. Jiao , Adv. Mater. 2017, 29, 1604540. 10.1002/adma.201604540 28151565

[25] M.‐L. Tsai , M.‐Y. Li , J. R. D. Retamal , K.‐T. Lam , Y.‐C. Lin , K. Suenaga , L.‐J. Chen , G. Liang , L.‐J. Li , J.‐H. He , Adv. Mater. 2017, 29, 1701168. 10.1002/adma.201701168 28650580

[26] K. W. Lau , Calvin , Z. Gong , H. Yu , W. Yao , Phys. Rev. B 2018, 98, 115427.

