# Supplementary material for: Multilayer Lateral Heterostructures of Van Der Waals Crystals with Sharp, Carrier–Transparent Interfaces
Source: Adv Sci (Weinh). 2021 Nov 23;9(3):2103830. doi: 10.1002/advs.202103830 (PMC8787400; doi:10.1002/advs.202103830)
Supplement: Supplementary file 1 — Supporting Information [file ADVS-9-2103830-s001.pdf]

ADVANCED SCIENCE
Open Access

Supporting Information

for *Adv. Sci.,* DOI: 10.1002/advs.202103830

Multilayer Lateral Heterostructures of Van der Waals Crystals with Sharp, Carrier-Transparent Interfaces

*Eli Sutter, Raymond R. Unocic, Juan-Carlos Idrobo, and Peter Sutter**

Supporting Information

Multilayer Lateral Heterostructures of Van der Waals Crystals with Sharp, Carrier-Transparent Interfaces

*Eli Sutter, Raymond R. Unocic, Juan-Carlos Idrobo, and Peter Sutter**

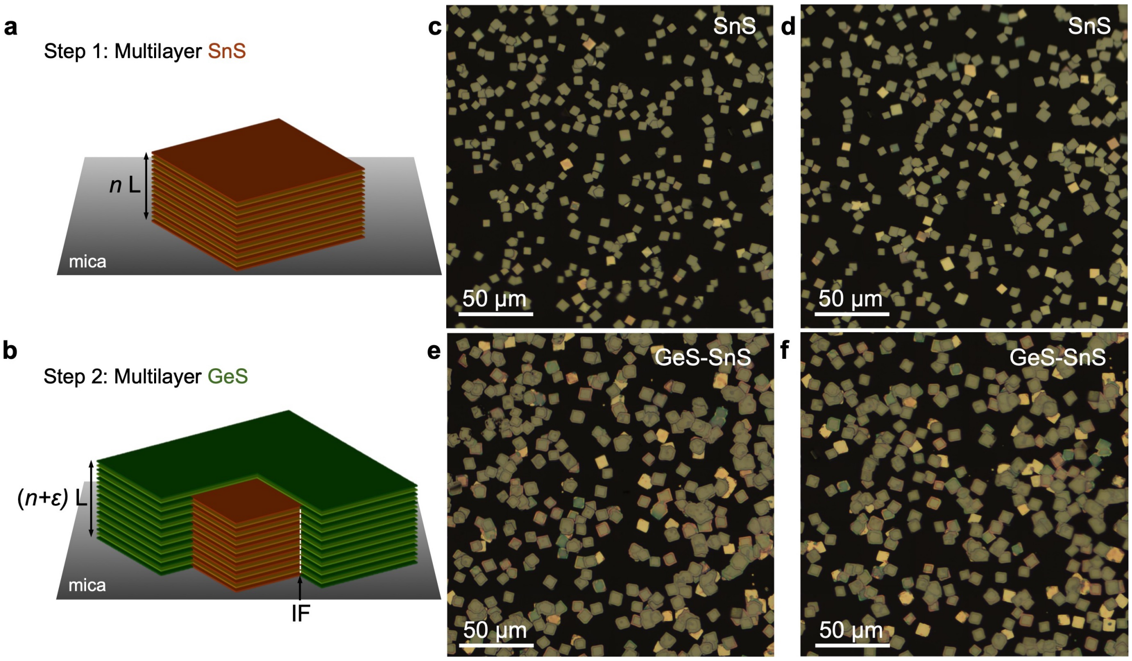

Figure S1 – Two-step vapor transport growth of multilayer lateral heterostructures. a. – b. Schematic of the two-step vapor transport growth process. **a.** The first growth step uses evaporation of a SnS precursor (source temperature: 650 °C) to produce dense ensembles of multilayer SnS flakes with typical sizes up to ~10 µm. **b.** In the second growth step, GeS vapor transport (Source temperature: 400 – 420°C; sample temperature: 300 – 320°C) encapsulates the SnS seeds to form multilayer lateral heterostructures covered by a thin GeS capping layer. **c. – d.** Optical microscopy of the mica substrate after the first growth step, showing an ensemble of large, faceted SnS flakes. **e. – f.** Optical microscopy of the same sample following the second vapor transport step, which converts each of the SnS seed flakes by preferential edge attachment of GeS into multilayer heterostructures.

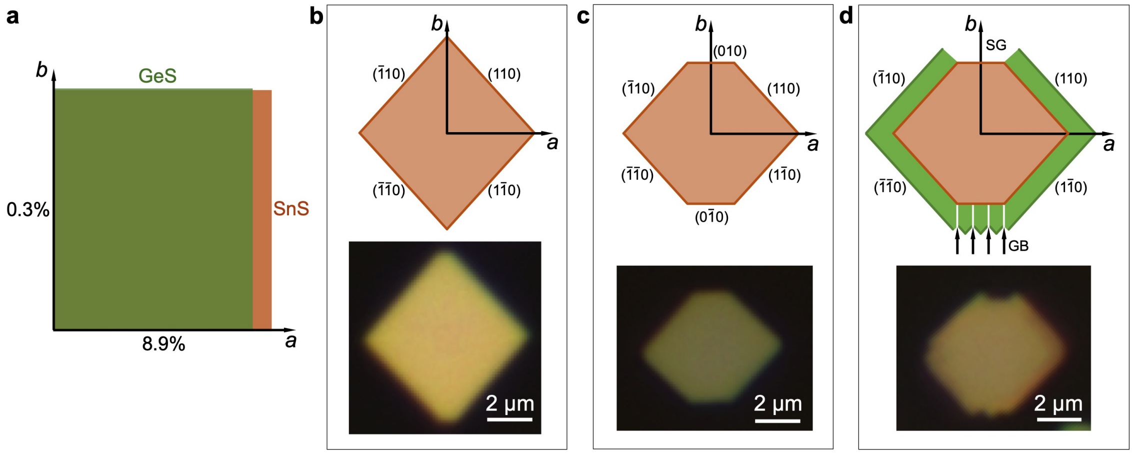

Figure S2 – Effects of the anisotropic lattice mismatch between SnS and GeS. a. Illustration of the in-plane unit cells of SnS and GeS. The lattice mismatch along the ***a***-axis is ~8.9%, whereas the mismatch along the ***b***-axis is only 0.3%. **b.** Schematic illustration (top) and optical image (bottom) of a mostly {110} faceted SnS flake (detail from Supporting Figure 1c) after the first (SnS) vapor transport growth step. **c.** Schematic illustration (top) and optical image (bottom) of a truncated SnS flake (detail from Supporting Figure 1c) after the first (SnS) vapor transport growth step. **d.** Schematic illustration (top) and optical image (bottom) of a multilayer heterostructure assembled by GeS growth onto a truncated SnS flake (detail from Supporting Figure 1e) following the second (GeS) vapor transport growth step. Note the suppressed GeS growth (SG) along some of the {010} facets and polycrystalline growth with grain boundary (GB) grooving and faceting along others (see also Figure 2).

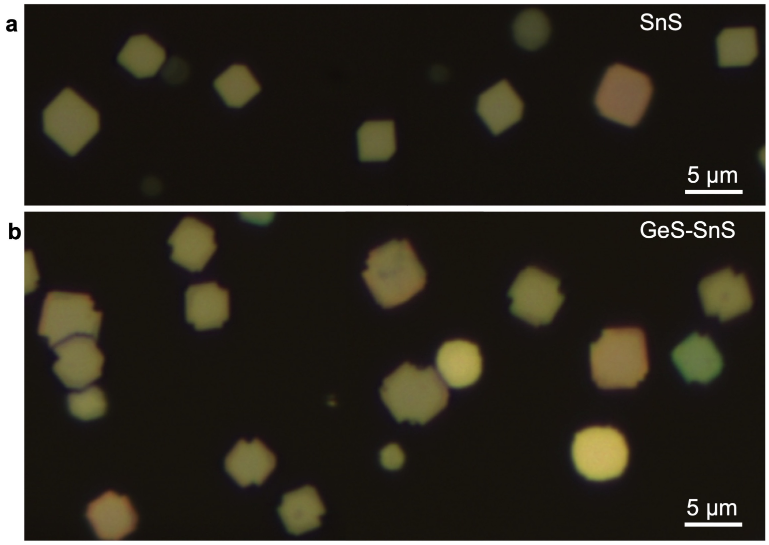

Figure S3 – Effects of the anisotropic lattice mismatch along the *a*-axis of SnS and GeS. a. Optical microscopy of an ensemble of truncated SnS flakes. **b.** Multilayer GeS-SnS heterostructures formed by GeS growth onto truncated SnS flakes, showing suppressed GeS growth in some of the corner regions due to the large lattice mismatch along the ***a***-axes of the orthorhombic layered crystals.

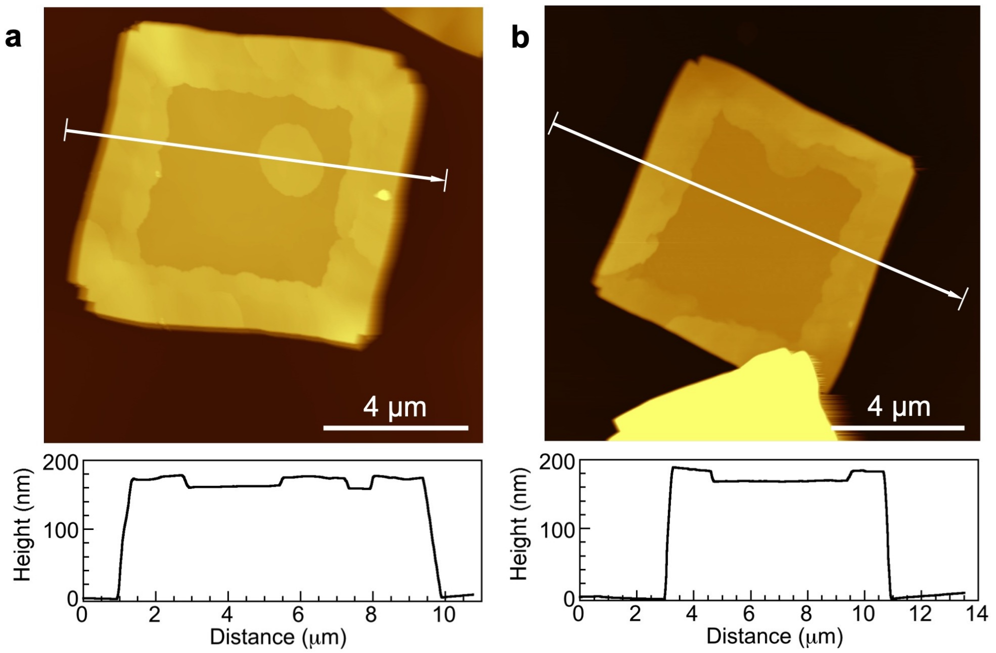

Figure S4 – Atomic force microscopy of typical multilayer GeS-SnS heterostructures. a. – b. AFM images (top) and height profiles (bottom) of two typical multilayer heterostructures. While the entire flakes are covered by a thin GeS capping layer, the GeS edge layer grows to somewhat larger thickness. Occasionally, GeS growth spirals atop the SnS seed crystals occasionally produce a locally thicker GeS cap (see panel a.).

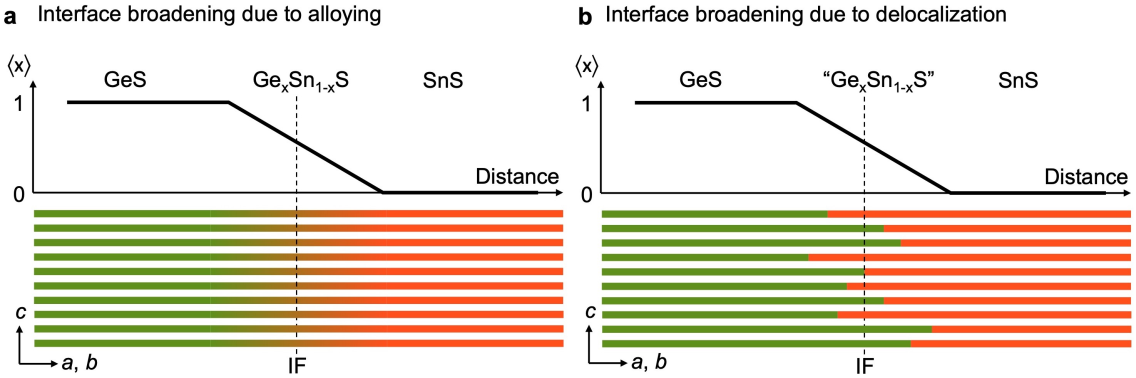

Figure S5 – Possible origins of interfacial broadening in multilayer lateral heterostructures of van der Waals crystals. a. Interface broadening due to intermixing or alloying between the components. **b.** Interface broadening due to delocalization, i.e., interface ‘roughness’ along the out-of-plane direction.

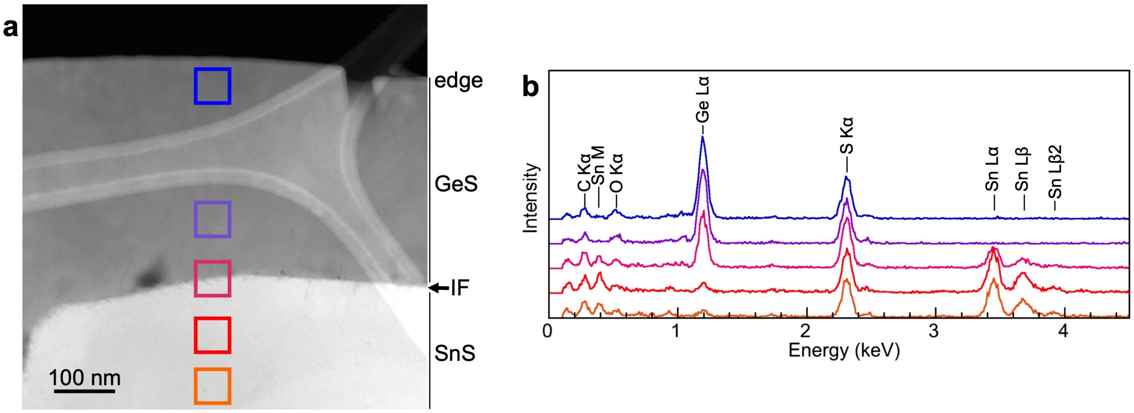

Figure S6 – EDS spectra within the SnS seed, GeS edge band, and at the lateral interface. a. HAADF-STEM image showing the same area of a multilayer lateral heterostructure depicted in Figure 4 of the main text. **b.** EDS spectra averaged within the areas marked by colored squares in panel a. Note the distribution of the main components, Sn, Ge, and S. The spectra show no elevated levels of contaminant species such as O and C at the lateral interface.

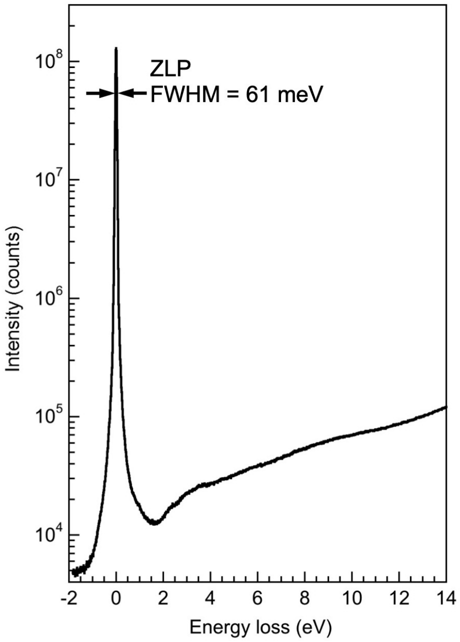

Figure S7 – STEM-EELS spectrum on the GeS side of the multilayer interface. Using monochromated STEM-EELS, a full width at half maximum (FWHM) or the zero-loss peak (ZLP) of 61 meV is obtained.

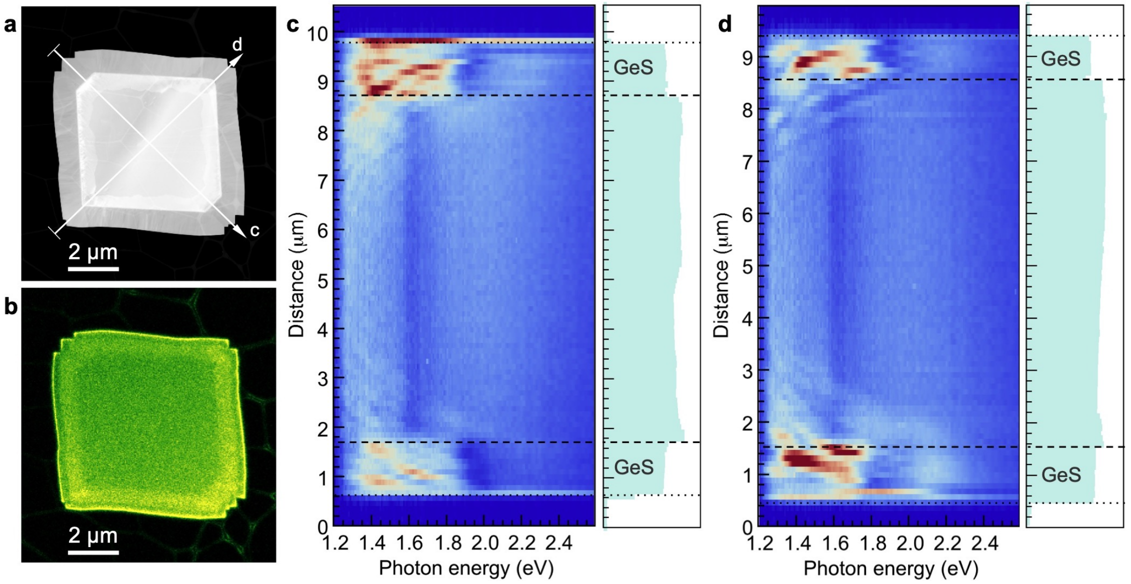

Figure S8 – STEM cathodoluminescence (STEM-CL) linescans along the diagonals of a multilayer GeS-SnS heterostructure. a. HAADF-STEM image of a typical heterostructure. **b.** Panchromatic CL map of the heterostructure shown in a. **c.** Diagonal linescan along the line marked ‘c’ in a. **d.** Diagonal linescan along the line marked ‘d’ in a. The right hand sides of panels c. and d. show the corresponding STEM intensity profiles.

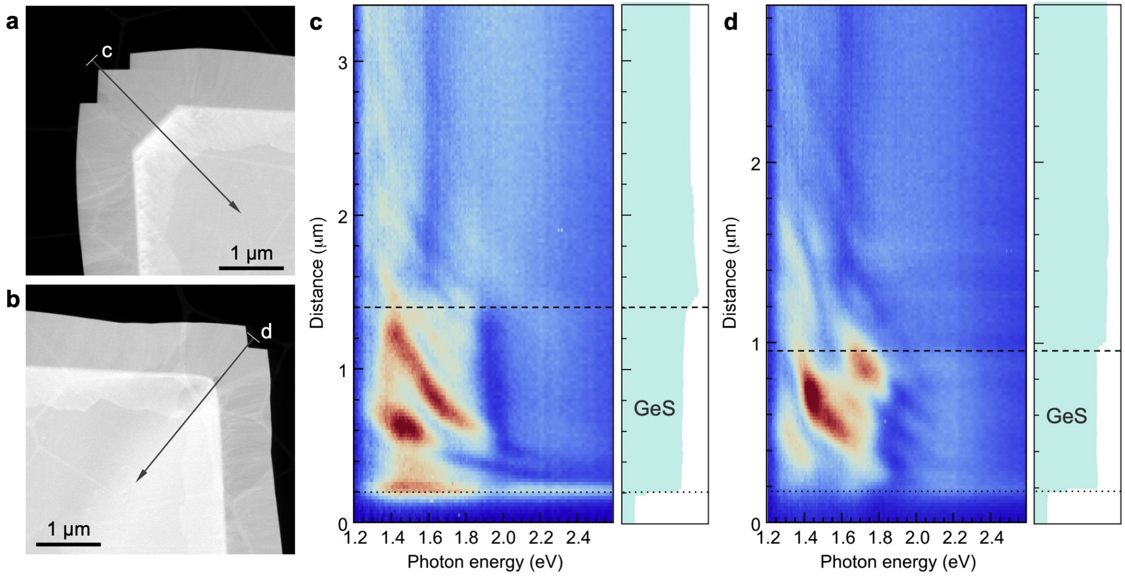

Figure S9 – STEM cathodoluminescence (STEM-CL) details near the corners of a GeS-SnS multilayer heterostructure. a. HAADF-STEM image obtained near the small (010) facet at one of the corners of a heterostructure. **b.** HAADF-STEM image obtained near the sharp (100) corner of the same heterostructure.**c.** Hyperspectral STEM-CL linescan along the line in a. **d.** Hyperspectral CL linescan along the line in b. The right hand sides of panels c. and d. show the corresponding HAADF-STEM intensity profiles.

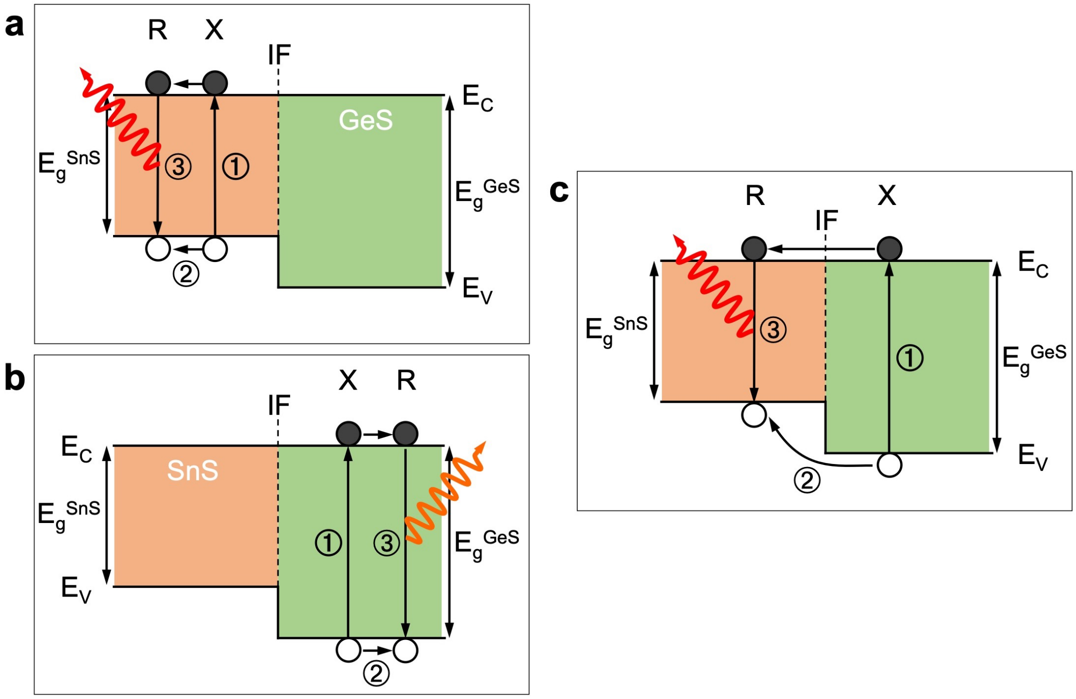

Figure S10 – Excitation, transport, and recombination of electron-hole pairs in lateral GeS-SnS heterostructures. a. Electron beam excitation (X), diffusion, and radiative recombination (R) on the SnS side of the multilayer lateral interface (IF). **b.** Electron beam excitation (X), diffusion, and radiative recombination (R) on the GeS side of the multilayer lateral interface (IF). **c.** Electron beam excitation (X) in GeS, followed by exciton transfer across the interface (IF) and radiative recombination (R) in SnS. While the processes shown in panels a. and b. produce luminescence consistent with the material at the location of the exciting electron beam, the process shown in panel c. gives rise to low-energy (SnS) luminescence for electron beam positions within GeS. Band offsets are shown as calculated by Malone and Kaxiras (Phys. Rev. B 87, 245312 (2013)) for interfaces between unstrained GeS and SnS.
